# The Expression and Roles of the Super Elongation Complex in Mouse Cochlear Lgr5+ Progenitor Cells

**DOI:** 10.3389/fncel.2021.735723

**Published:** 2021-10-01

**Authors:** Yin Chen, Ruiying Qiang, Yuan Zhang, Wei Cao, Leilei Wu, Pei Jiang, Jingru Ai, Xiangyu Ma, Ying Dong, Xia Gao, He Li, Ling Lu, Shasha Zhang, Renjie Chai

**Affiliations:** ^1^Jiangsu Provincial Key Medical Discipline (Laboratory), Department of Otolaryngology Head and Neck Surgery, Drum Tower Hospital Clinical College of Nanjing Medical University, Nanjing, China; ^2^State Key Laboratory of Bioelectronics, Jiangsu Province High-Tech Key Laboratory for Bio-Medical Research, School of Life Sciences and Technology, Southeast University, Nanjing, China; ^3^Department of Otorhinolaryngology, Head and Neck Surgery, The Second Hospital of Anhui Medical University, Hefei, China; ^4^Department of Otolaryngology, The First Affiliated Hospital of Wenzhou Medical University, Wenzhou, China; ^5^Co-innovation Center of Neuroregeneration, Nantong University, Nantong, China; ^6^Institute for Stem Cell and Regeneration, Chinese Academy of Sciences, Beijing, China; ^7^Beijing Key Laboratory of Neural Regeneration and Repair, Capital Medical University, Beijing, China

**Keywords:** super elongation complex (SEC), inner ear, expression, proliferation, differentiation

## Abstract

The super elongation complex (SEC) has been reported to play a key role in the proliferation and differentiation of mouse embryonic stem cells. However, the expression pattern and function of the SEC in the inner ear has not been investigated. Here, we studied the inner ear expression pattern of three key SEC components, AFF1, AFF4, and ELL3, and found that these three proteins are all expressed in both cochlear hair cells (HCs)and supporting cells (SCs). We also cultured Lgr5+ inner ear progenitors *in vitro* for sphere-forming assays and differentiation assays in the presence of the SEC inhibitor flavopiridol. We found that flavopiridol treatment decreased the proliferation ability of Lgr5+ progenitors, while the differentiation ability of Lgr5+ progenitors was not affected. Our results suggest that the SEC might play important roles in regulating inner ear progenitors and thus regulating HC regeneration. Therefore, it will be very meaningful to further investigate the detailed roles of the SEC signaling pathway in the inner ear *in vivo* in order to develop effective treatments for sensorineural hearing loss.

## Introduction

Hearing loss occurs mainly due to noise exposure, aging, ototoxic drugs, and genetic factors (Sun et al., 2017). There were around 466 million people worldwide with disabling hearing loss in 2020, and the World Health Organization [WHO] (2019) estimates that by 2050 over 900 million people will have disabling hearing loss. Deafness has become a major global health problem, and sensorineural hearing loss is the most common type of hearing impairment (Youm and Li, 2018). However, due to the lack of effective drugs and a non-invasive method for targeted delivery of drugs to the inner ear, the treatment options for sensorineural hearing loss are limited (Mittal et al., 2019). Cochlear hair cells (HCs) in adult mammals lose the ability to regenerate, thus hearing deficits caused by HC loss are permanent (Warchol et al., 1993; Ryan, 2003; Cox et al., 2014; Xu et al., 2017; Chen et al., 2019). Therefore, induction of HC regeneration after injury by stimulating quiescent inner ear progenitor cells has been a main focus of auditory research in recent years.

The super elongation complex (SEC) is extremely important in the transcriptional elongation checkpoint control stage of transcription and is composed mainly of P-TEFb (positive transcription elongation factor), ELL (11–19 lysine-rich leukemia gene) family proteins, AFF (AF4/FMR2) family proteins, ENL (11–19 leukemia), AF9 (ALL1-fused gene from chromosome 9), and many other transcription factors (Luo et al., 2012b). P-TEFb and ELL are RNA polymerase II (Pol II)-related elongation factors (Shilatifard et al., 2003). AFF family proteins act as transcriptional activators with a positive action on RNA elongation (Melko et al., 2011). ENL and AF9 are homologous, and they can connect the SEC to RNA Pol II-related factors (He et al., 2011). P-TEFb is composed of cyclin-dependent kinase 9 (CDK9) and cyclin T (CycT), and it promotes the transition into productive elongation by phosphorylating RNA polymerase II (Peng et al., 1998). It has been reported that the SEC plays an important role in regulating mouse embryonic stem cell proliferation and differentiation (Lin et al., 2011), and mis-regulation of the SEC leads to the uncontrolled regulation of gene expression during the differentiation of embryonic stem cells, which results in a variety of diseases such as acute lymphoblastic leukemia, cerebellar ataxia, and diffuse midline glioma (Lin et al., 2011; Dahl et al., 2020). ELL3, one of the key factors of the SEC, can protect differentiated cells from apoptosis by promoting the degradation of p53, enhancing the differentiation of mouse embryonic stem cells, and regulating the proliferation and survival of embryonic stem cells (Ahn et al., 2012). However, the roles of the SEC in the inner ear remain unclear.

Flavopiridol is a semi-synthetic flavonoid that has been used in the treatment of acute myeloid leukemia (Zeidner and Karp, 2015), chronic lymphocytic leukemia (Wiernik, 2016), and other chronic diseases. Flavopiridol binds directly to CDK9, which is a component of P-TEFb, and inhibits its kinase activity (Chao et al., 2000). In turn, P-TEFb, as an important component of the SEC, can activate RNA polymerase II and transcriptional elongation (Hnisz et al., 2013). Thus, the most common method for blocking SEC function is to directly inhibit CDK9 with flavopiridol (Morales and Giordano, 2016), and we used flavopiridol to inhibit the function of the SEC as previously reported (Lin et al., 2011).

Recent studies have shown that Lgr5+ supporting cells (SCs) are inner ear progenitors and that they have the ability to regenerate new HCs in the neonatal stage (Shi et al., 2012). The activation of Wnt/β-catenin signaling and inhibition of Notch signaling can induce Lgr5+ progenitors to regenerate Myo7a+ HCs (Chai et al., 2012; Korrapati et al., 2013; Mizutari et al., 2013), and several recent studies have also shown that Lgr5+ progenitors can be regulated by many other factors and signaling pathways such as Shh, Foxg1, and Hippo (Gregorieff et al., 2015; Chen et al., 2017; Zhang et al., 2020). However, the regeneration efficiency of Lgr5+ progenitors is still very limited, which suggests that there are other factors or signaling pathways involved in the HC regeneration process. Because the transcription extension stage is the main stage of gene expression regulation, transcriptional regulation of developmental regulatory genes is the core link between embryonic stem cell differentiation and organ formation (Smith and Shilatifard, 2010; Levine, 2011). Therefore, we speculate that the SEC may also play important roles in cochlear progenitor cells.

Here we measured the expression of the key SEC factors AFF1, AFF4, and ELL3 in the neonatal mouse cochlea, the function of SEC inhibitor flavopiridol in House Ear Institute-Organ of Corti 1 (HEI-OC1) cell line, and we assessed the proliferation and differentiation ability of Lgr5+ progenitors after treatment with the SEC inhibitor flavopiridol. Our results suggest important roles for the SEC in Lgr5+ progenitors *in vitro*, and further *in vivo* studies need to be done to elucidate the roles of the SEC in the inner ear. These studies will form the experimental basis for using cochlear progenitors to regenerate functional HCs in order to treat patients with sensorineural hearing loss.

## Materials and Methods

### Experimental Animals

Lgr5-EGFP-Ires-CreERT2 (Lgr5-EGFP) mice (Barker et al., 2007) (Jackson Laboratory, Stock No. 00887) and FVB mice used as wide-type mice were raised in a comfortable environment with suitable temperature and light and fed with standard laboratory food and water *ad libitum*. We are approved by the Animal Care and Use Committee of Southeast University and were consistent with the National Institutes of Health Guide for the Care and Use of Laboratory Animals. All the operations were carried out in accordance with the procedures.

### RNA Extraction and Reverse Transcription-Polymerase Chain Reaction

About 20 wild-type mouse cochleae were dissected to extract total RNA, which was reverse transcribed into cDNA with the cDNA Synthesis Kit (Thermo Fisher Scientific, K1622). Gene expression was measured by reverse transcription-polymerase chain reaction (RT-PCR) with GAPDH as the endogenous reference gene. The RT-PCR conditions were as follow for a total of 35 cycles: initial denaturation at 95°C for 15 s, denaturation at 95°C for 15 s, annealing at 60°C for 60 s, and extension at 72°C. The primers were as follows: GAPDH: (F) 5′-AGG TCG GTG TGA ACG GAT TTG-3′; (R) 5′-TGT AGA CCA TGT AGT TGA GGT CA-3′; AFF1: (F) 5′-GAA GGA AAG ACG CAA CCA AGA-3′; (R) 5′-TAG CTC ATC GCC TTT TGC AGT-3′; AFF4: (F) 5′-ATG AAC CGT GAA GAC CGG AAT-3′; (R) 5′-TGC TAG TGA CTT TGT ATG GCT CA-3′; ELL3: (F) 5′-GAC CAG CCT CCT GAT GCT AAG-3′; (R) 5′-GCC ACC ATT AGT GCC CTC TTG-3′.

### Western Blotting

About 10 cochleae from postnatal day (P)3 mice were dissected in order to extract proteins. GAPDH was used as the reference protein. The primary antibodies were anti-AFF1 (Sigma-Aldrich, #SAB2106246), anti-AFF4 (Santa Cruz, #sc135337), and anti-ELL3 (Abcam, #ab67415). Peroxidase-conjugated goat anti-rabbit (Life, A-31572) and goat anti-mouse (Invitrogen, A21202) were used as the secondary antibodies. The gray levels were measured by Image-J.

### Cell Culture

HEI-OC1 cells were cultured in Dulbecco’s modified Eagle’s medium (DMEM) with 10% fetal bovine serum and 1% ampicillin at 37°C and 5% CO_2_. The cells were divided into two groups. The experimental group was treated with flavopiridol (AbMole M1710) at the concentration of 10 μM. Control cells were treated with dimethyl sulfoxide (DMSO) in the same culture medium. After 12-h culture, cells were treated with 0.25% trypsin/ethylene diamine tetraacetic acid (EDTA) and then ultrasonicated (Bioruptor^TM^ UCD-200) for CDK9 kinase detection.

### Cyclin-Dependent Kinase 9 Kinase Assay

HEI-OC1 cells with or without flavopiridol treatment were used after ultrasonication to detect the CDK9 activity by using CDK9 Cyclin K Kinase Assay kit (Promega, V4104) and ADP-Glo Kinase Assay kit (Promega, V6930). To initiate the CDK9 reaction, CDK9 substrate PDKtides and adenosine triphosphate (ATP) were added into each group for 120 min at room temperature to produce adenosine diphosphate (ADP) according to the manufacturer’s instruction (Promega, #TM313). And then ADP-Glo Reagent was added for 40 min at room temperature to deplete the remaining ATP. The Kinase Detection Reagent was added to convert the ADP produced at the first step to ATP with luminescence. Finally, the luminescence was recorded by BioTek CYTATION 5 (Integration time 1 s) to determine the CDK9 activity in each sample. The relative light units were calculated to represent the activity of CDK9.

### Isolation of Lgr5+ Progenitors *via* Flow Cytometry

About 50–60 cochleae were isolated from P0 to P3 Lgr5-EGFP mice and then treated with 0.125% trypsin/EDTA (Invitrogen, 25200114) at 37°C. Trypsin inhibitor (10 mg/ml, Worthington Biochem) was added after 10 min to terminate the reaction. The trypsinized cochleae were pipetted up and down 80–100 times to obtain single cells, and the cells were then filtered through a 40 μM cell strainer (BD Biosciences, 352340). Dissociated cells were sorted on a flow cell sorter (BD FACS Aria III). The EGFP+ cells were collected as Lgr5+ progenitors for further *in vitro* cell culture experiments.

### Sphere-Forming Assay and Differentiation Assay

Sorted Lgr5+ cells were cultured in DMEM/F12 medium at a density of 2 cells/μl (200 cells per well) for 5 days for sphere forming. The formula of DMEM/F12 medium was the same in previous study (Zhang et al., 2020). Spheres were identified with the Live Cell Imaging System and quantified using Image J. For differentiation, cells were cultured in the DMEM/F12 medium described above at a density of 20 cells/μl (2,000 cells per well) for 10 days. EdU [10 μM (Invitrogen, C10420)] was added to label proliferating cells from day 4 to day 7. Flavopiridol (AbMole, M1710) was added to the experimental group from day 1 to day 10 at a concentration of 10 μM, while DMSO was added to the control group. Differentiated neurospheres were analyzed by immunofluorescent staining.

### Immunofluorescent Staining

The cochleae were dissected in cold Hanks Balanced Salt Solution (HBSS) in order to prevent protein degradation and then fixed with 4% paraformaldehyde (PFA) for 1 h at room temperature. *In vitro* cultured neurospheres were also fixed with 4% PFA for 1 h at room temperature. After washing with phosphate buffered saline with tween (PBST) three times, the cochleae or neurospheres were blocked with blocking solution for 1 h at room temperature and then incubated overnight at 4°C with primary antibodies. The primary antibodies used were anti-Myosin7a (Myo7a; Proteus Bioscience, #25-6790; 1:1,000 dilution), anti-Sox2 (1:400 dilution), anti-AFF1 (1:400 dilution), anti-AFF4 (1:50 dilution), and anti-ELL3 (1:400 dilution). After washing again three times, the cochleae or neurospheres were further incubated with secondary antibodies (Invitrogen, A21131, A21124) diluted 1:400 in PBT2 for 1 h at room temperature. After washing three times, the cochleae or neurospheres were mounted on slides with anti-fade fluorescence mounting medium (DAKO, S3023). Images were captured by Zeiss LSM 710 confocal microscope and analyzed by Image J software.

### Tissue Embedment

The P40 temporal bones were dissected and put in 4% PFA to be shaken for 1–2 h and sit overnight at 4°C. Later, the temporal bones were put in 0.5 M EDTA for decalcification for 2 days. After washing with PBST three times, the temporal bones were transferred into 15% sucrose solution, vacuum for 1 h, 4°C overnight. Afterward, the temporal bones were put in 20% sucrose solution, vacuum for 1 h, then transferred to 30% sucrose solution, vacuum 1 h, 4°C overnight. Then, the temporal bones were put into a 1:1 solution of 30% sucrose in optimum cutting temperature (OCT) medium (Sakura 4583), vacuum for 1 h, overnight at 4°C. The following day, temporal bones were put in a 3:7 solution of 30% sucrose in OCT medium, vacuum for 1 h, then the 3:17 solution of 30% sucrose in OCT medium, vacuum for 1 h, and lastly the 100% OCT medium (adjust position as round window and ellipse window are toward on the ground), vacuum for 1 h, 4°C overnight. For the last step, the temporal bones were put in 100% OCT into vacuum for 1 h adjust position, then in cryostat (Microm HM525) for a 20-min quick-freeze, and restored in −80°C. For slicing, adjust the cryostat half an hour in advance; secondly, adjust the blade temperature and internal temperature to −20°C. The selected sections were stained using the method described above.

### Statistical Analysis

All the data in this research are presented as means ± SEM, and all experiments were repeated at least three times. All statistical analyses were performed in GraphPad Prism 5. *P*-values were calculated using a two-tailed, unpaired Student’s *t*-test, and a *p*-value < 0.05 was considered statistically significant.

## Results

### AFF1, AFF4, and ELL3 Are Expressed in the Cochlea

**FIGURE 1:**
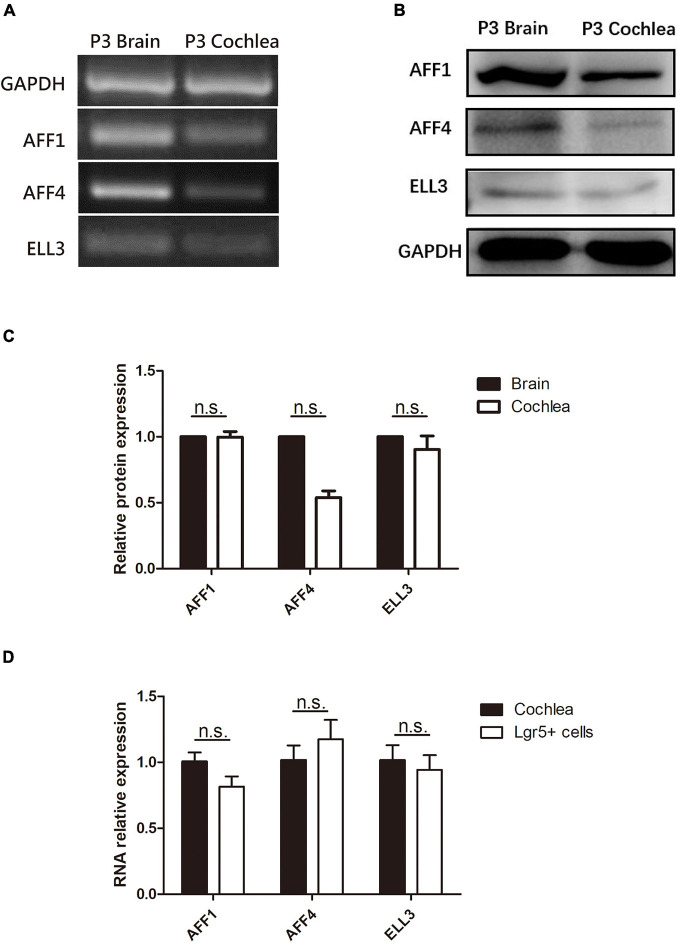
The expression of AFF1, AFF4, and ELL3 in the neonatal mouse cochlea. **(A,B)** The mRNA and protein expression of AFF1, AFF4, and ELL3 in P3 mouse cochleae were detected by RT-PCR **(A)** and western blotting **(B)**, respectively. **(C)** The gray levels comparison of western blot. **(D)** The mRNA expression of AFF1, AFF4, and ELL3 in Lgr5+ cells. Brain samples of P3 mice were used as the positive control, and GAPDH was used as the internal reference. n.s., not significant.

**FIGURE 2:**
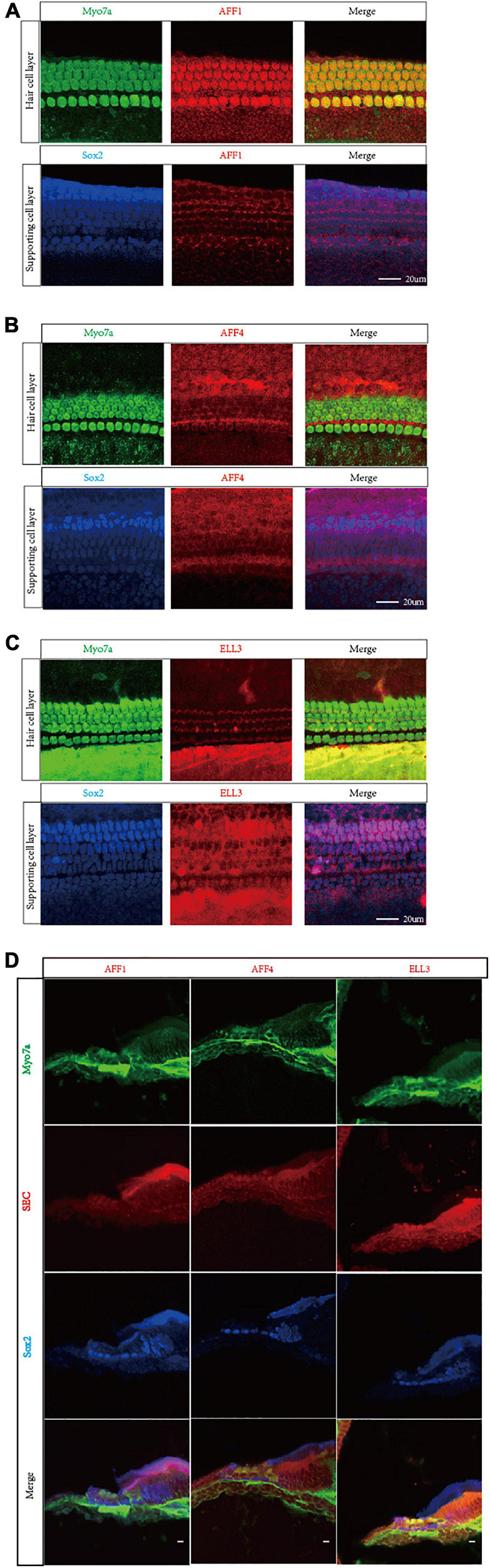
Immunofluorescent staining of AFF1, AFF4, and ELL3 in the mouse cochlea. **(A–C)** The whole-mount basilar membrane of P3 was immunostained by antibodies against AFF1 **(A)**, AFF4 **(B)**, and ELL3 **(C)**. **(D)** Frozen sections of P40 were immunostained by antibodies against AFF1, AFF4, and ELL3. Myo7a and Sox2 were used to label hair cells (HCs) and supporting cells (SCs), respectively. Scale bar = 20 μM.

We first measured the expression of the three key SEC subunits AFF1, AFF4, and ELL3 by RT-PCR (Figure 1A) and Western blotting analysis (Figures 1B,C), and we found that AFF1, AFF4, and ELL3 were all highly expressed in the cochlea. Moreover, we measured the expression of AFF1, AFF4, and ELL3 in Lgr5+ cells (Figure 1D), and we found those three expressions in both cochlea and Lgr5+ cells were similar. We further studied the expression pattern from both the obverse and lateral sides of AFF1, AFF4, and ELL3 in the cochlea of P3 and P40 mice and found that AFF1, AFF4, and ELL3 were all expressed in the cochlear HCs and SCs (Figures 2A–D). However, the immunostaining intensities of these three subunits in the SCs were weaker than in the HCs.

### Flavopiridol Treatment Inhibited the Activity of Cyclin-Dependent Kinase 9 in House Ear Institute-Organ of Corti 1 Cells

**FIGURE 3:**
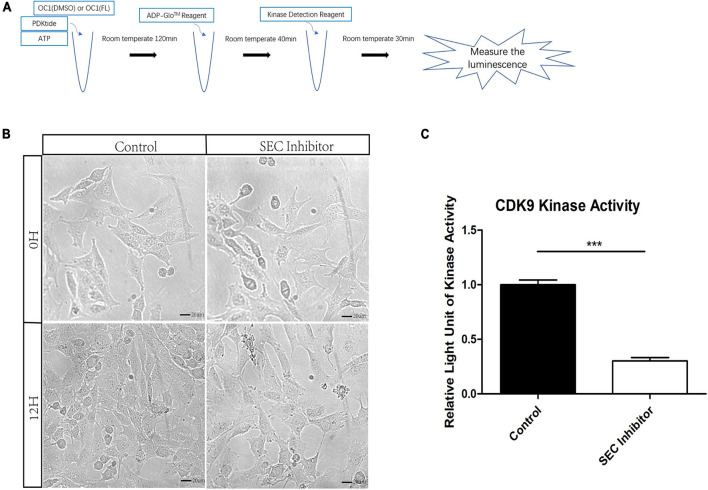
The activity of cyclin-dependent kinase 9 (CDK9) in HEI-OC1 cells before and after Flavopiridol treatment. **(A)** HEI-OC1 cells after ultrasonication were incubated with CDK9 substrate PDKtides and ATP for 120 min at room temperature. The ADP which was consumed during CDK9 kinase assay was converted to ATP and could be quantitated by luciferase assay. **(B)** Images of HEI-OC1 cells before and after 10 μM Flavopiridol treatment. DMSO was added as control treatment in the Control group. Scale bar = 20 μM. **(C)** Fold change of CDK9 kinase activity in HEI-OC1 cells with or without Flavopiridol treatment. *n* = 3, ***p < 0.001.

Flavopiridol has been reported to be an inhibitor of CDK9 which is an indispensable part of SEC and the low level of its kinase activity prevents the recruitment of other elongation factors in SEC (Peng et al., 1998). However, the function of flavopiridol has not been verified in inner ear. Here, we used ADP-Glo Kinase Assay to detect the activity of CDK9. After the kinase reaction, the remaining ATP was depleted and the ADP was converted to luminescent ATP (Figure 3A). HEI-OC1 cells were cultured for 3 days, and then treated by 10 μM flavopiridol which was diluted in the culture medium for 12 h. Images of cells were taken before and after flavopiridol treatment (Figure 3B). After flavopiridol treatment, the number and diameter of cells visually decrease in comparison with the control group with no change in shapes. The luminescent ATP was recorded and the relative light units was calculated to represent the activity of CDK9 (Figure 3C). The results showed that flavopiridol could also function as the CDK9 inhibitor to inhibit SEC activity in HEI-OC1 cells.

### Flavopiridol Treatment Decreased the Sphere-Forming Ability of Lgr5+ Progenitors *in vitro*

**FIGURE 4:**
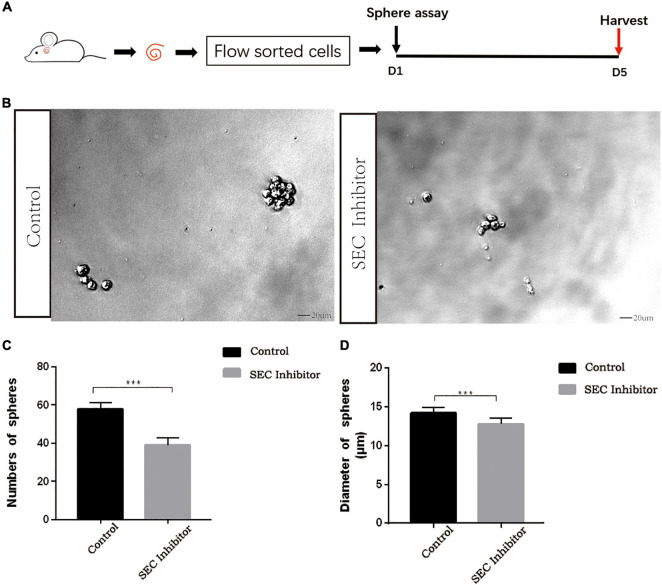
The sphere-forming assay of Lgr5+ progenitors after inhibition of the super elongation complex (SEC). **(A)** Schematic of the sphere-forming assay. The cochleae of neonatal Lgr5-EGFP-CreER mice were trypsinized for FAC sorting, and the sorted Lgr5+ progenitors were cultured *in vitro* for 5 days to form spheres with or without 10 μM flavopiridol treatment. **(B)** The spheres (indicated by black arrows) formed by Lgr5+ progenitors with 10 μM flavopiridol added as the SEC inhibitor. DMSO was added as control treatment in the Control group. Scale bar = 20 μM. **(C,D)** Quantification of the sphere number per well **(C)** and the sphere diameter **(D)**. *n* = 3. ***P < 0.001.

Flavopiridol was previously used to inhibit SEC transcription activity (Lin et al., 2011). Here we also used flavopiridol to inhibit SEC activity in Lgr5+ progenitors in order to determine whether the SEC plays roles in the proliferation and differentiation ability of Lgr5+ progenitors. In order to determine the effect of the SEC on the sphere-forming ability of Lgr5+ progenitors, Lgr5+ cells were isolated from Lgr5-EGFP mice by flow cytometry and then cultured *in vitro* for 5 days to form spheres with or without 10 μM flavopiridol treatment (Figures 4A,B). The flavopiridol treatment decreased both the number (Figure 4C) and diameter of the spheres (Figure 4D), which suggested that inhibition of the SEC could decrease the sphere-forming ability and proliferation ability of Lgr5+ progenitors *in vitro*.

### No Difference Was Observed in the Differentiation Assay After Flavopiridol Treatment

**FIGURE 5:**
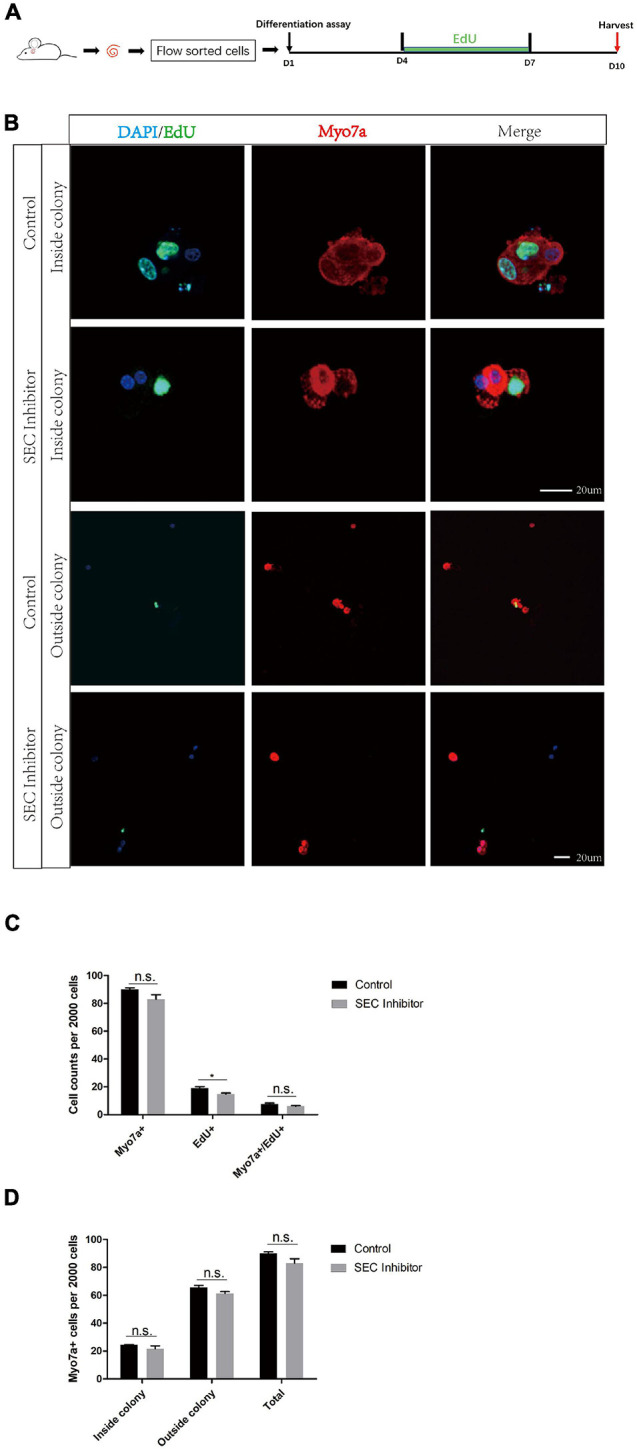
The differentiation assay of Lgr5+ cells after inhibition of the super elongation complex (SEC). **(A)** Schematic of the differentiation assay. The cochleae of neonatal Lgr5-EGFP-CreER mice were trypsinized for FAC sorting, and the sorted Lgr5+ progenitors were cultured *in vitro* for 10 days with or without 10 μM flavopiridol. EdU was added during day 4–7 to label proliferating cells. **(B)** Immunofluorescence images of colonies and single cells formed from Lgr5+ progenitors with 10 μM flavopiridol added as the SEC inhibitor. DMSO was used as the control treatment in the Control group. Myo7a and Sox2 were used to label hair cells (HCs) and supporting cells (SCs), respectively, and EdU was used to label proliferating cells. Scale bar = 20 μM. **(C,D)** Quantification of the total numbers of Myo7a+ and EdU+ cells **(C)** and the number of Myo7a+ cells inside and outside the colonies **(D)**. *n* = 3. n.s., not significant. *P < 0.05.

In order to further evaluate the effect of the SEC on the differentiation ability of Lgr5+ progenitors, we isolated Lgr5+ cells by flow cytometry and cultured them *in vitro* for the differentiation assay with or without 10 μM flavopiridol treatment (Figure 5A). The cells were immunostained with Myo7a, EdU, and DAPI (Figure 5B), and the Myo7a+ cells and EdU+ cells inside and outside the colonies were quantified. There were more EdU+ cells in the flavopiridol treatment group than in the control group (Figure 5C), while the numbers of Myo7a+ cells were almost the same in the flavopiridol treatment group and the control group (Figure 5D). These results suggested that inhibition of the SEC did not affect the differentiation ability of Lgr5+ cells *in vitro*.

In summary, our results showed that the key SEC factors AFF1, AFF4, and ELL3 are all highly expressed in the cochlea. And we verified that flavopiridol could inhibit SEC by inhibiting CDK9 activity in HEI-OC1 cell line. We used flavopiridol as an SEC inhibitor to investigate the effect of the SEC on the proliferation and differentiation ability of Lgr5+ progenitors and found that the number and diameter of spheres of Lgr5+ progenitors were both decreased after SEC inhibitor treatment, while the differentiation ability of Lgr5+ progenitors was not affected. Therefore, the SEC appears to promote the proliferation ability of Lgr5+ progenitors, but not their differentiation ability.

## Discussion

Irreversible damage to HCs in the mammalian cochlea is the main cause of sensorineural hearing loss. Previous studies have reported that Lgr5+ cells are cochlear progenitors with the ability to regenerate HCs (Shi et al., 2012), and it has been documented that the SEC plays an important role in the process of gene transcription and extension and that it is necessary for the differentiation of mouse embryonic stem cells (Lin et al., 2011). Therefore, we speculated that the SEC also plays an important role in the proliferation and differentiation of mouse cochlear progenitors. Besides, it has also been reported that AFF proteins and ELL proteins increase the diversity and regulatory potential of the SEC family in mammals (Luo et al., 2012a). Furthermore, AFF1 and AFF4 are the backbones of the SEC (Mück et al., 2016) and ELL3 has the ability to associate with other translocation partners of the SEC (Lin et al., 2011). Thus we chose to investigate the expression of AFF1, AFF4, and ELL3 in the inner ear. Our results showed that AFF1, AFF4, and ELL3, were all highly expressed in the cochlea and that the SEC inhibitor flavopiridol could induce the proliferation of Lgr5+ progenitors *in vitro*, but not their differentiation. This study thus provides an experimental foundation for the clinical application of HC regeneration for treating hearing loss.

The SEC is known to be widely expressed in most tissues, and it plays important roles during development (Kapushesky et al., 2010). However, its expression in the inner ear has not been studied. We found that AFF1, AFF4, and ELL3 were highly expressed in the cochlea, and this suggests that the SEC functions in the inner ear. Furthermore, we found that AFF1, AFF4, and ELL3 were all expressed in the cochlear HCs and SCs by immunostaining, but the expression of SEC proteins in SCs was lower than that in HCs. In addition to its role in Lgr5+ progenitors studied in our research, the SEC likely plays an essential role in cochlear HCs. However, due to the lack of studies of the SEC in the inner ear and the lack of mouse models, the specific role of the SEC in HCs awaits further study.

Flavopiridol, a potent inhibitor of CDK9, is reported to inhibit transcription (Blagosklonny, 2004; Lee and Zeidner, 2019). CDKs combine with cyclins to play important roles in transcription and stem cell self-renewal (Lim and Kaldis, 2013), and P-TEFb (composed of CDK9 and CycT) is an indispensable part of the SEC that phosphorylates RNA polymerase II and thus activates transcription elongation of important genes involved in cell proliferation and survival (Zhu et al., 1997; Zeidner and Karp, 2015). Flavopiridol can inhibit the function of the SEC by inactivating CDK9 (Chao and Price, 2001), and thus we chose flavopiridol as the SEC inhibitor as previously reported (Lin et al., 2011).

In addition, our results suggest that inhibition of the SEC by flavopiridol could reduce the sphere-forming ability and proliferating cell number of Lgr5+ progenitors in the differentiation assay, which is consistent with previous reports that AFF1 promotes leukemia cell proliferation (Fioretti et al., 2019), that AFF4 enhances the sphere-forming capacity and tumor-initiation capacity in head and neck squamous cell carcinoma (Deng et al., 2018), and that ELL3 stimulates the proliferation and stem cell properties of breast cancer cells (Ahn et al., 2013).

ELL3 has also been shown to promote the differentiation of mouse embryonic stem cells by regulating the epithelial-mesenchymal transition and apoptosis (Ahn et al., 2012), and the overexpression of AFF1 impairs the differentiation of mesenchymal stem cells, while overexpression of AFF4 enhances their differentiation (Zhou et al., 2017). In our results, inhibition of the SEC did not affect the differentiation ability of Lgr5+ progenitors *in vitro*, which might be because of the different regulatory roles of these three proteins in cell differentiation.

The different roles of SEC in the proliferation and differentiation ability of Lgr5 progenitors might have been expected. SEC was first systematically studied for being associated with infant acute lymphoblastic and mixed lineage leukemia (Lin et al., 2010). Then it has since been studied in other neoplastic models (Hashizume et al., 2014; Narita et al., 2017; Liang et al., 2018). While transcriptional regulation is a complex process, it has been sure that the role of SEC is essential in transcriptional elongation. The SEC incorporates the CDK9 to promote rapid transcriptional elongation facilitates cell growth (Dahl et al., 2020). However, speaking of the differentiation ability of SEC, totally different results were found concerning the different components of this complex. Our paper first studied the differentiation ability of the total complex and found that the whole SEC cannot change the differentiation in Lgr5 progenitors. There is a certain possibility that this finding may also apply to other stem cells. The detailed mechanisms behind this need further exploration.

## Conclusion

In conclusion, we show here that the SEC is expressed in cochlear HCs and SCs in neonatal mice and that the SEC can induce the proliferation ability of Lgr5+ progenitors but not their differentiation. Our study thus provides new candidates for regulating inner ear progenitor cells as a step toward HC regeneration.

## Data Availability Statement

The raw data supporting the conclusions of this article will be made available by the authors, without undue reservation.

## Ethics Statement

The animal study was reviewed and approved by the Animal Care and Use Committee of Southeast University, the National Institutes of Health, and the Guide for the Care and Use of Laboratory Animals.

## Author Contributions

YC, HL, LL, SZ, and RC conceived and designed the experiments and wrote the manuscript. YC, RQ, YZ, WC, YD, LW, PJ, JA, and XM performed the experiments. YC, RQ, YZ, WC, XG, LL, SZ, and RC analyzed the data. All authors read and approved the final manuscript.

## Conflict of Interest

The authors declare that the research was conducted in the absence of any commercial or financial relationships that could be construed as a potential conflict of interest.

## Publisher’s Note

All claims expressed in this article are solely those of the authors and do not necessarily represent those of their affiliated organizations, or those of the publisher, the editors and the reviewers. Any product that may be evaluated in this article, or claim that may be made by its manufacturer, is not guaranteed or endorsed by the publisher.
